# Child Health in the Peruvian Amazon: Prevalence and Factors Associated with Referred Morbidity and Health Care Access in the City of Iñapari

**DOI:** 10.1155/2015/157430

**Published:** 2015-11-10

**Authors:** Maria Gabriela Silva Guimarães, Athos Muniz Braña, Humberto Oliart-Guzmán, Fernando Luiz Cunha Castelo Branco, Breno Matos Delfino, Thasciany Moraes Pereira, Saulo Augusto Silva Mantovani, Antonio Camargo Martins, Ana Paula Santos, José Alcântara Filgueira-Júnior, Alanderson Alves Ramalho, Andreia da Silva Guimarães, Cristieli Sérgio de Menezes Oliveira, Thiago Santos de Araújo, Carlos Hermógenes Manrique de Lara Estrada, Nancy Arróspide, Mônica da Silva-Nunes

**Affiliations:** ^1^Centro de Ciências da Saúde e do Desporto, Universidade Federal do Acre, Campus Universitário, BR 364, km 04, Bairro Distrito Industrial, 69920-900 Rio Branco, AC, Brazil; ^2^Dirección Regional de Salud de Madre de Dios, Avenida Ernesto Rivero N° 475, Puerto Maldonado, Peru; ^3^Instituto Nacional de Salud, Cápac Yupanqui 1400, Jesus María, 15072 Lima 11, Peru

## Abstract

*Introduction*. Children under 5 years of age are more susceptible to developing morbidities such as diarrhea, respiratory infections, anemia, and malnutrition. The objective of the study is to evaluate the prevalence of reported morbidities in this age group in the city of Iñapari (Peru) and the access to health services in this municipality. *Methods*. Data collection using interviews that assessed socioeconomic and demographic conditions, child morbidity, and access to health services was performed in 2011. Statistical analysis was performed using SPSS 13.0. *Results*. Regarding morbidities that occurred during lifetime, 39.8% reported previous anemia and intestinal parasite infection. About 53.7% of the children reported any type of morbidities in the last 15 days before interview, being most frequent respiratory symptoms (38.9%), diarrhea (23,4%), and fever (23,1%). Only 63.1% of those reporting recent morbidities sought health care. These morbidities were associated with precarious sanitation and lack of infrastructure, the presence of other comorbidities, and poor access to health services. *Conclusion*. The main referred morbidities in Amazonian Peruvian children were diarrhea, respiratory symptoms, anemia, and vomiting. Incentives and improvements in the health and sanitation conditions would be important measures to improve the quality of life of the Amazonian child population.

## 1. Introduction

Children under five years of age are an important population group in regard to their physiological and psychosocial development [1]. As a consequence, they are more susceptible to the development of various morbidities, such as diarrhea [2, 3], respiratory infections [2, 4], anemia [5], and malnutrition [4, 6].

Most cases of acute diarrhea and mortality from this disease occur in children under five years of age [2]. This increased prevalence is due to a combination of poor hygiene habits [2], poor sanitation [2, 7], and unfavorable socioeconomic conditions [2, 7].

Respiratory infections are another frequent morbidity in this group [2]. Children suffer the greatest impact from air pollutants [8], increasing the risk of respiratory illness. Additionally, contact with other children in daycare facilities promotes the acquisition of respiratory diseases due to overcrowding and exposure to possible infectious agents.

In relation to anemia, children under five years are the most vulnerable because of their increased need for iron [9], higher nutritional requirements during this period of life [10], their transition in the types of food they intake [10], and a higher susceptibility of the acquisition of intestinal parasites [11].

Malnutrition is an indicator of both health and nutrition [6]. The main causes of malnutrition are inadequate intake of nutrients [5] and infectious diseases [6], specifically respiratory and gastrointestinal diseases.

Since this age group is very susceptible to morbidity, attention must be paid to ensure that these children have access to health services. Provision of prenatal and childbirth care is effective measures in reducing infant mortality [12], as well as access to health care in both routine situations and emergencies.

Peru, located in South America and bordered to the east by Brazil, was ranked 82nd by the Human Development Index (HDI) in 2013 [10]. This is an index that considers wealth, education, and health as proxies for human development. In 2011, 36.19% of visits to Peru's health services were from children under 11 years of age [11]. According to the 2010 National Demographic and Health Census of Peru [4], 17.1% of Peruvian children under five years of age showed symptoms of acute respiratory infection and 14.9% had acute diarrheal disease.

Anemia is also one of the most prevalent nutritional problems in Peru [13]. In 2010, 50.3% of children under three years of age were anemic, with 56.6% being from rural areas [14]. Malnutrition is also a large health problem in Peru [4]. According to the National Institute of Statistics and Informatics (INEI), the prevalence of chronic malnutrition in Peruvian children under five years was 23.2% in 2010 [15].

This study aims to evaluate the prevalence of reported morbidities in children under five years of age in the municipality of Iñapari (Peru) and their access to health services in the municipality.

## 2. Materials and Methods

**Figure 1:**
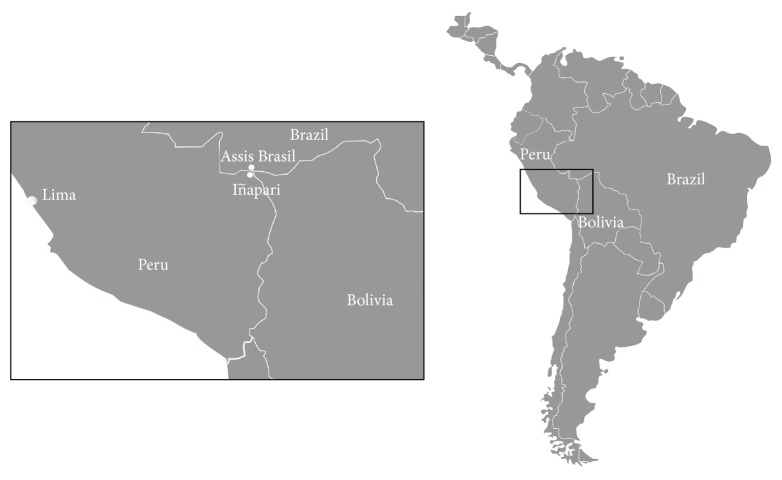
Map of Iñapari, Peru.

This study was conducted in the urban area of Iñapari, a municipality located in the Peruvian Amazon, in the triborder region between Brazil (Assis, Brazil), Bolivia (Bolpebra), and Peru. Iñapari is a district of the Amazon province of Tahuamanu and is located in the Madre de Dios Department (Figure 1). The estimated city population for 2010 was 1,434 people [16].

The study selected as a criterion for inclusion children under 5 years of age, because they are at greater risk of health inequalities and a greater susceptibility to the development of diseases because of that.

Children eligible for the study were identified using a census of households carried out by the health workers of Iñapari, which identified children under the age of five years living in the urban area of the city in 2011.

Data collection for the study took place between January and February 2011 using structured interviews that evaluated the socioeconomic and demographic conditions of the family (family income, maternal/guardian education level, presence of government scholarship, and maternal/guardian paid work in the last 30 or 90 days), household environmental conditions (type of housing construction, predominant material on the floor of the house, presence of electricity, presence of paved street, and type of toilet in the household), peridomestic environmental conditions (garbage collection, flooding of the peridomestic area during rain, and presence of open sewers near the house), water supply and treatment (water supply for domestic use, presence of running water inside the house, and type of water treatment), the child's demographic information (age, sex, and ethnicity), reported infant morbidity (morbidities in the last 15 days, morbidities in the last 12 months, morbidities ever in life, and hospitalization cases), and access to health services (health care, medication). Interviews were conducted by medical students who spoke Spanish, supervised by a Peruvian medical doctor and a Peruvian biologist with a M.S. degree in public health. Interviews were performed with the biological mother, and when she was not present, the biological father or the guardian of the child was interviewed. A few variables contained missing values (one or two unknown responses). The variable “is the child living with the biological father?” was a sensitive question and therefore resulted in a larger number of absences of response. Interviews were conducted by medical students who spoke Spanish, supervised by a Peruvian medical doctor and a Peruvian biologist with a M.S. degree in public health.

For statistical analysis, a database was created using SPSS Version 19.0 software (SPSS Inc., Chicago, IL). The ANOVA test was used to compare means, and Pearson's Chi-square test or Fisher's exact test was used to compare frequencies or proportions at the level of 5%.

The study was approved by the Ethics Committee for Human Research of Acre Federal University (Brazil) and of the Instituto Nacional de Salud (Lima, Peru) (processes 23107.008153/2010-92 UFAC and 2010-CI-59-INS). Informed consent was obtained from the participants in the study or from the parents or legal guardians of the minors prior to the interviews.

## 3. Results

The study was conducted with all children living in the urban area of the city. There were 108 children under five years of age living in the urban area of Iñapari. Of these, 46.3% were females and 53.7% were males, with a mean age of 2.11 years (standard deviation of 1.36 years and median of 2.24 years). All children were included in the study. Regarding ethnicity, 7.4% declared themselves white, 2.8% indigenous, 38.9% brown (“pardos”), and 50.9% mestizo. Almost all children (93.5%) were living with their biological mother, and 95.6% lived with their biological father. Maternal education was as follows: 68.9% of the mothers had more than eight years of schooling and 1.9% had no schooling (Table 1).

Houses were made predominantly of wood (82.4%), with latrines (67.6%) and unpaved streets or undefined streets (76.9%). Almost half (47.7%) of households reported being in flood-prone areas and 22.2% had open sewage in the peridomestic environment (Table 1).

In the study group, 39.8% of children reported having had some type of morbidity during their lifetime. The most frequently reported morbidities were anemia (23.1%) and intestinal parasites (“worms,” 18.5%), and less frequent were wheezing (3.7%) and asthma (2.8%). As for the occurrence of these morbidities in the last 12 months, anemia (20.6%) and intestinal parasites (13%) were still the most frequent morbidities, and less frequent were wheezing (3.7%) and asthma (1.9%) (Table 2).

The prevalence of children who had ever been consulted by a doctor or nurse was 89.8%, and the prevalence of consultation in the last 12 months was 81.5%. Only 16.7% of children were reported to have been hospitalized at least once in their life.

In relation to morbidities occurring in the last 30 days, 56.5% of the children referred to having had some morbidity. The most common were fever (28.7%) and diarrhea (22.2%), and the least common were nausea (3.7%) and shortness of breath (3.7%) (Table 3). Regarding the previous 15 days prior to the interview, 53.7% of children reported some type of morbidity. Respiratory symptoms were the most frequent (38.9%) and included wheezing (4.6%), runny nose (22.2%), dry cough (14.8%), productive cough (16.7%), and sore throat (18.5%). Other morbidities were diarrhea (23.4%), fever (23.1%), and vomiting (15.7%) (Table 4).

About 63.1% of children who became ill used the health service, and 97.2% of these had access to health care. A drug prescription was filled in 91.1% of cases, and in these cases the drug was purchased from the public health care system (37.5%), purchased by the patient at the pharmacy (53.1%), or in a few cases purchased at the pharmacy by the health service (9.3%) (Table 5).

For 36.8% of children who did not seek health care for their morbidities, the main reasons for such behavior were that parents did not find it necessary (68.2%), there was lack of time to seek the health service or self-medication was practiced (31.8%), or parents thought there would be no available health care (4.5%).

In Iñapari, 18.5% of the children reported the presence of worms in their lifetime and 13% reported this condition in the last 12 months. There was an association between infection and age (*P* = 0.006, Chi-square test), showing that, in children older than two years, the prevalence of worms was higher. There was also an association between previous intestinal parasites and no piped water into the household (*P* = 0.038, Fisher's test) and failure to consult with and/or little follow-up by the health service, confirming the hypothesis of little access to antiparasitic drugs.

Respiratory symptoms in the last 15 days prior to the interview were associated with the presence of diarrhea in the same period of time (*P* = 0.001, Fisher's test), maternal smoking (*P* = 0.037, Chi-square test), the type of street the house was located on (brick or asphalt) (*P* = 0.022, Fisher's test), and the type of sidewalk (brick or cement) (*P* = 0.022, Fisher's test). All other variables were tested but did not show association. Variables associated with the occurrence of respiratory symptoms are shown in Table 6.

Diarrhea in the last 15 days prior to interview was also associated with the presence of fever (*P* = 0.018, Fisher's test), vomiting in the last 15 days (*P* = 0.002, Chi-square test), presence of “stomachache” in the last 30 days (*P* = 0.002, Chi-square test), and lack of maternal paid work over the past 90 days (*P* = 0.045, Fisher's test).

When analyzing the presence of diarrhea in a longer period of time (in the 30 days preceding the interview), it was possible to detect an association with other symptoms: fever (*P* = 0.003, Fisher's test), vomiting (*P* = 0.047, Chi-square test), and weakness in the last 30 days (*P* = 0.014, Chi-square test). Some socioeconomic variables were also associated, such as absence of maternal paid work over the past 90 days (*P* = 0.05, Fisher's test), lack of water piped into the household (*P* = 0.026, Fisher's test), not boiling water before consumption (*P* = 0.016, Fisher's test), and having a latrine or no toilet at home (*P* = 0.049, Fisher's test) (Table 7). All other variables were tested but did not show association. Variables associated with the occurrence of diarrhea in the last 30 days are shown in Table 7.

Factors associated with hospital admission anytime in life are shown in Table 8. Having been hospitalized was significantly more frequent in females, children older than two years, low maternal education, and poor prior access to health care. Children whose mothers did not have morbidities during pregnancy were admitted for hospitalization more frequently than those who did.

## 4. Discussion

The main recently referred morbidities in this study were diarrhea, fever, vomiting, and respiratory complaints. In relation to morbidity that occurred sometime in their lives, the most frequent were anemia and intestinal parasites.

According to the World Health Organization [17], Brazil and Peru are among the Latin American countries with the highest prevalence of anemia in preschool children. According to the National Institute of Statistics and Informatics, the prevalence of anemia in children under five years of age in Peru was 37.7% in 2010 [4]. The prevalence of anemia was higher in rural areas (45.7%) [4] than in urban areas of the country (33.0%) [4]. In the Department of Madre de Dios, where Iñapari is located, the prevalence of anemia in children was 44.7%, while it was much lower (28.3%) in the Department of Lima [4].

Grantham-McGregor et al. [18] performed a review of 33 studies and found that school and preschool children were more susceptible to the development of anemia and that anemic children had lower income level in relation to nonanemic children.

Zanin et al. [19] found a prevalence rate of anemia of 35.9% in Brazilian children from Minas Gerais, which was associated with iron deficiency, parasitic infections, being at risk of or being a low length/height-for-age, and lower retinol intake. All of these are well-known risk factors for anemia in children.

A review of studies from recent, large population health surveys, carried out in 11 French-speaking African countries (Benin, Burkina Faso, Cameroon, Congo Brazzaville, Ivory Coast, Gabon, Guinea, Mali, Niger, Democratic Republic of Congo, and Senegal) identified factors associated with anemia [20]. Anemia (Hb < 11 g/dL) was found in 72.4% of the children (60.2–87.8%), with no gender difference but a slightly lower incidence in older children (62% at age of 4-5 years versus 85% at age of 9 months). In these countries, anemia was associated with location (rural areas), income (lower quintile), and low maternal education.

In Acre, Brazil, the prevalence of anemia in children under five years of age living in small communities was 57.3% in 2005, and it was primarily associated with nutritional deficiency in this age group [17]. In other municipalities of Acre, including the neighboring city of Assis, Brazil, the prevalence of anemia in children younger than five years of age was 29.2% in 2003, being higher in children under two years of age and also associated with diarrhea [21]. These are similar rates to those found in Iñapari. The prevalence of anemia during the course of life in children under five years of age was 23.1%, and the prevalence in the last 12 months was 20.6%. This is a lower frequency than appointed by National Institute of Statistics and Informatics for Madre de Dios (44.7%) [4]. This difference may be explained because our data is based on referred morbidities, and there might be undiagnosed cases among our study population since anemia is a silent disease in some cases, especially in those who do not seek health services on a regular basis. Anemia was not associated with age in this study, as it was in other studies in Acre [17, 21].

Children are most vulnerable to developing parasitic intestinal infections [22, 23], especially when in contact with other people at home or school, due to a lack of hygiene [2]. Intestinal parasites are also associated with poor socioeconomic [17, 23, 24] and sanitation conditions [23, 24]. According to the National Institute of Statistics and Information (Peru), in 2013 the proportion of people with basic sanitation in the region of Madre de Dios was only 43.8% [25], while those with access to potable water were only 93.1% [22]. These conditions are worse than those found in the Department of Lima, the capital of Peru, where basic sanitation was available to 90.3% of children from this age group and access to potable water was available to 99.3% [22, 23], thus demonstrating a higher exposure of the population of Madre de Dios to poor sanitation and related infectious diseases. In Iñapari, 67.6% of the children had a latrine at home, and 22.2% were living in houses near open sewage. Such associations are reported in children from other developing countries as well [2, 26].

Cha et al. [27] conducted a trial in rural areas of Ghana, to estimate the effects of water treatment in the incidence of diarrhea, adjusting the analysis in the presence of sanitation. The adjusted prevalence ratio of diarrhea in the intervention compared with the control communities was 0.82 (95% CI 0.71–0.96) for Krachi West, 0.95 (0.86–1.04) for Krachi East, and 0.89 (0.82–0.97) for both districts. This study provides a basis for a better approach to water quality interventions.

Data from the National Institute of Statistics and Informatics [4] show that in Peruvian children under five years of age, the prevalence of diarrhea in the last 15 days prior to the interview was 14.9% in 2010, with a higher prevalence in children living in the region of the Peruvian Amazon [4]. In Madre de Dios, the prevalence of diarrhea in the last 15 days in children was 22.4% in 2010 [4], a smaller prevalence than that found in the Department of Lima or on the Peruvian coast (13.9%) [4]. Therefore, the prevalence found in the present study in Iñapari (23.4%) in 2011 is similar to that reported for the whole Peruvian Amazon and higher than reported on the Peruvian coast. One possible cause of this discrepancy may be related to the growing urbanization of the Amazon without adequate infrastructure and sanitation [28].

Diarrhea was mainly associated with unfavorable socioeconomic conditions and poor hygiene habits, such as drinking untreated water. Children who consumed no boiled water had more diarrhea (32.7%) than those that consumed boiled water (13.6%). The same association was found in other Peruvian children [4]. Ochoa et al. [3] also found that in Peruvian children diarrhea was associated with age, breastfeeding, and natural immunity obtained by previous infections.

In Tanzania, diarrhea was associated with unfavorable socioeconomic conditions, younger age, and lack of toilet at home [24]. In Ghana, data from 3466 children showed that diarrhea was associated with household wealth quintiles, child sex and age, being the poorer, male children, and younger children more likely to have diarrhea [29].

A study with 348,706 children from 40 nations found that the prevalence of acute diarrhea was 14% [30]. The main factors associated with child diarrhea were poor household wealth and mother's lack of education. Other factors associated with diarrhea were female sex of the child, age of the child, immunization status, maternal age, and working status of the mother.

A large case-control hospital-based study conducted in Brazil [31] showed that lack of adequate excreta disposal (PAF = 12%), untreated drinking water (PAF = 11%), and a history of previous hospitalization due to acute diarrhea (PAF = 21%) were the main factors attributed to hospitalization due to diarrhea. Low socioeconomic conditions, no public water supply, crowding, and low weight-for-age were of less but still significant importance [31].

A study with 5,828 indigenous Brazilian children found an overall prevalence of recent diarrhea of 23.5%, being the highest rate in the North of the country (38.1%). Higher risk of diarrhea was observed among younger children and those who had less maternal schooling, lower household socioeconomic status, undernutrition (weight-for-age deficit), presence of another child with diarrhea in the household, and occurrence of upper respiratory infection [32].

The diarrheal process may be associated with other clinical manifestations such as fever, vomiting, and abdominal pain, as shown by other studies [33–35]. These clinical manifestations can suggest possible infectious agents involved in gastroenteritis [36], such as* Rotavirus*,* E. coli*,* Shigella*, and* Salmonella* species.

Intervention studies focusing on education about how to prevent infant diarrhea were conducted with 600 mothers in Egypt [37]. Results showed that knowledge of mothers about diarrhea (etiological factors and preventive measures) had improved significantly after the intervention and that health and nutrition education sessions were successful in improving mothers' knowledge regarding preventive measures and management of diarrhea. A meta-analysis of 22 randomized clinical trials conducted in low- and middle-income countries found that hand washing promotion probably reduces diarrhea episodes in both child daycare centers in high-income countries and among communities living in LMICs by about 30% [38]. Such measurements could be implemented by health care workers in other countries, such as Peru, in order to reduce the incidence of diarrhea in young children.

The second most prevalent morbidity in Iñapari was respiratory disease. In a study conducted in Brazilian nurseries, Pedraza et al. [2] showed that acute respiratory disease is a group of important diseases in children, as they are more vulnerable due to the immaturity of the immune system and their rapid growth. Heymann et al. [36] comment that, in the United States, hospital admission still continues to be a significant health care problem for children.

In Iñapari, the prevalence of respiratory symptoms in the last 15 days was 38.9%. In the country as a whole, the prevalence of respiratory disease in the last 15 days in children under five years of age in 2010 was 17.1%, in the Madre de Dios region it was 15.4%, and it was 18.3% in the region of Lima, suggesting a relationship between respiratory symptoms and the degree of urbanization of the area [4]. This difference in prevalence may also be the result of differences in the concept of respiratory disease among the studies. In the approach adopted by INEI [4] in Peru, the concept of respiratory disease was reported as illness associated with the presence of fast and/or troubled breathing, while, in the study in Iñapari, respiratory symptoms were defined as the occurrence of one or more symptoms such as wheezing, runny nose, cough (dry or productive), or sore throat.

Brauer et al. [39] found association of respiratory symptoms of children to environmental pollution in The Netherlands. In this study, the prevalence of respiratory symptoms was different from one region to another suggesting that regions with greater development and urbanization had higher incidence of children with respiratory symptoms, corroborating the findings in Peruvian children described above.

The association found in Iñapari between child respiratory symptoms and having tobacco users at home has been documented in previous studies [34, 36]. Gonçalves-Silva et al. [40] showed that not only can parental smoking be associated with child respiratory symptoms, but also smoking by any other resident of the household. In Iñapari, the frequency of respiratory symptoms in children whose mothers were smokers was 77.8%, while, in the study by Gonçalves-Silva et al. [40], it was 65.6%.

A review of studies from 11 French-speaking African countries also found that respiratory disease and diarrhea are common in children under 5 years of age. In the study countries, acute respiratory diseases and diarrhea affected 6.2 and 15.6% of children aged between 6 and 59 months, respectively [20].

In India, a community-based cross-sectional study found an overall prevalence of acute respiratory infections of 59.1%, which was associated with overcrowding (adjusted odds ratio (AOR) = 1.492), urban residence (AOR = 2.329), and second birth order (AOR = 0.371) [41].

Data from the Peruvian Ministry of Health [42] showed a hospitalization rate of 3.03 per 100 inhabitants in the general population in 2010. Influenza (flu) and pneumonia accounted for the largest amount of hospitalization cases in that year [43]. In Madre de Dios, in the Peruvian Amazon, intestinal infections prevailed in relation to influenza and pneumonia as a cause of hospitalization in the general population in the same year [44]. In Iñapari, hospitalization of children under five years of age was mainly due to diarrhea and other intestinal diseases, confirming the data obtained by the Ministry of Health in the region.

Shay et al. [45] estimated that 84,000 to 144,000 hospitalization cases due to lower respiratory tract disease occur annually in the USA in children under 5 years of age.

In the study of Quigley et al. [46], conducted in the UK, approximately 12% of children in the first eight months of age were hospitalized at least once. The most common causes of hospitalization were infection of the lower respiratory tract (3.2%) and diarrhea (1.1%). The study demonstrated a related protection factor to breastfeeding for hospitalization of protection related to injury.

Factors associated with child hospitalization in Iñapari were related to child age and sex, socioeconomic characteristics, and access to health care. Children whose mothers had no formal education or low education were more likely to have been hospitalized. Macedo et al. [47] also found that child hospitalization increased when maternal education was low. In Iñapari, male children had a higher frequency of hospitalization (26.3%), a finding that was reported in other studies such as Macedo et al. [47], with 53.3%, and Caetano et al. [48], with 56.9%.

Also, the frequency of hospitalization was higher in children older than 2 years (26.3%) than in those younger than 2 years (5.9%). This could be explained by the fact that as time goes by, there are more chances to get sick. The association between age and hospitalization was also described in other studies Bittencourt et al. [49] found that hospitalization was higher in children younger than six months (60.3% in public hospitals and 47.3% in private hospitals), while Caetano et al. [48] found that hospitalization was higher in children younger than 1 year (47.7%). Differences in methodology and population characteristics can explain these differences in results.

A study in rural Tanzania showed that children aged two years and older were more likely than children under one year of age to receive care at home, rather than to receive care at the health facility. Also, children aged two years and older were less likely to experience morbidity than children under one year [35].

Caetano et al. [48] describe a frequency of 56.9% of child hospitalization in children younger than 5 years, being the main causes of respiratory and parasitic diseases.

In other Latin American countries, diarrhea and respiratory diseases are also a public health problem in childhood. In Haiti, respiratory diseases accounted for 9183 (29%) hospitalization cases and 301 (17%) deaths between 2011 and 2013. Children aged 6–23 months had the highest percentage of hospitalization cases attributable to respiratory diseases (38%), whereas children aged 36–47 months had the highest proportion of deaths attributable to respiratory diseases (37%). Diarrheal diseases accounted for 8063 (26%) hospitalization cases and 224 (13%) deaths. Children aged 6–11 months had the highest percentage of diarrhea-associated hospitalization cases (39%) and deaths (29%) [50].

The occurrence of morbidity during pregnancy was inversely associated with child hospitalization. About 27.3% of children with no history of maternal morbidity during pregnancy were hospitalized at some point during life, while only 9.8% of those without maternal history of gestational morbidity had a hospitalization. This could be explained by a modified maternal behavior in child care after a gestational morbidity, leading to improved maternal care or more access to health services during pregnancy and after birth.

Children with poor health access after birth were also more likely to have a hospitalization. About 26.7% of those without a medical appointment in the previous year had hospitalization, against only 9.5% of those who attended a medical appointment in the previous year. Hospitalization cases were more frequent in children who seek health care services only when sick or who never sought health services.

Access to health care was high in Iñapari when the child was sick but lower for routine consultations. It is known that health service usage usually varies according to the degree of economic development of an area or region [51], and it can also be different in urban and in rural areas [52]. According to Pêssoa [53], consultations and physical exams are more complete in Iñapari than in the neighboring Brazilian city of Assis, but the working conditions for health care personnel and the diagnostic infrastructure are worse, with low salaries and lack of diagnostic exams. Another important difference is that in Peru access to consults and medication within the health service require payment [53], while in Brazil these are free.

This study has some strengths and weaknesses. It has the merit of being a population-based census on child health performed in the Peruvian Amazon, which is usually an understudied type of population because of the difficult physical access. Interviews were performed in Spanish by Peruvian researchers, and one of them was living in the Peruvian Amazon, so it was performed by personnel that were used to the habits and local culture, facilitating the interviews. The limitations of the study are that since it was a small community, the number of children was small and some associations may have not been identified. Also, because it was based on referred morbidities, there may be some degree of recall bias which could not be avoided.

## 5. Conclusion

This study showed that the main morbidities in children referred to the Iñapari municipality were related to diarrheal morbidity, respiratory symptoms, anemia, and vomiting. There was an association of these morbidities referred to as low socioeconomic conditions, precarious sanitation, and the presence of other comorbidities. Measurements to reduce morbidity and promote child health in such small Peruvian Amazonian communities would be improvements in sanitary infrastructure, water treatment, educational programs on child health for targeted families, and a more comprehensive health care with lower costs. Another important point is that free care is not always provided in Peru, which may hinder access to health care for some children. Health policies that target free universal care for infants at risk may help promote child health in this part of the Amazon.

## Figures and Tables

**Table 1:** Epidemiological characteristics of children aged under 5 years, Iñapari, 2011.

Variables (*n* = 108)	*N*	%
*Characteristics of the child*		
Sex		
Male	58	53.7
Female	50	46.3
Mean and median age		
Mean	2.11 Years	—
Median	2.24 Years	—
Age		
<12 months	29	26.9
≥12 and <24 months	22	20.4
≥24 and <36 months	26	24.1
≥36 and <48 months	20	18.5
≥48 and <60 months	11	10.2
Ethnicity		
White	8	7.4
Indigenous	3	2.8
Brown (“Parda”)	42	38.9
Mestizo	55	50.9
Maternal figure taking care of the child		
Biological mother	101	93.5
Grandmother	6	5.6
No maternal figure	1	0.9
Years of maternal education^*∗*^		
0	2	1.9
1–4	6	5.7
5–8	25	23.6
>8	73	68.9
Living with biological father^#^		
Yes	87	82.9
No	18	17.1

*Parental characteristics*		
Paternal paid work over the past 30 days^*∗∗∗∗*^		
No	6	6.0
Yes	94	94.0
Paternal paid work over the past 90 days^*∗∗∗∗*^		
No	8	8.0
Yes	92	92.0
Maternal paid work over the past 30 days		
No	78	72.9
Yes	29	27.1
Maternal paid work over the past 90 days		
No	77	72.6
Yes	29	27.4
*Domestic and peridomestic environment*		
Type of household construction		
Predominantly made of brick	19	17.6
Predominantly made of wood	89	82.4
Presence of open sewage near house		
No	84	77.8
Yes	24	22.2
Type of street		
Soil or no street	83	76.9
Brick or asphalt	25	23.1
Type of toilet		
Flushed toilet	35	32.4
Latrine or no toilet	73	67.6
Susceptibility of household to flooding during rain^*∗∗∗*^		
No	56	52.3
Yes	51	47.7
Piped water		
No	9	8.3
Yes, inside home	70	64.8
Yes, out of home	29	26.9
Domestic waste disposal		
Public garbage collection	97	89.8
Buried or burned	6	5.6
Played in the environment (open area or river)	5	4.6
House floor		
Cement, brick ceramic tile	35	32.4
Wood or land	75	67.6
Source of domestic water		
Only public system	83	76.9
Dwelling or public system	23	21.3
Other sources	2	1.9
Treatment of drinking water		
Untreated	9	8.3
Mineral untreated	28	25.9
Boiled and/or filtered and/or chlorinated	61	56.5
Only chlorinated	10	9.3
Presence of electric power		
No	7	6.5
Yes	101	93.5
Receipt of benefits		
No	43	39.8
Yes	65	60.2
Family income^##^		
Up to half minimum wage	2	1.9
More than half minimum wage	102	98.1

^*∗*^ *n* = 106, ^*∗∗∗*^ *n* = 107, ^*∗∗∗∗*^ *n* = 100, ^#^ *n* = 105, and ^##^ *n* = 104.

**Table 2:** Frequency of referred morbidities in children under 5 years of age, Iñapari, 2011.

Variables (*n* = 108)	*n*	(%)
Morbidities		
Lifetime	43	39.8
Previous 12 months	36	33.3
Anemia		
Lifetime	25	23.1
Previous 12 months^*∗*^	22	20.6
Intestinal parasites (“worms”)		
Lifetime	20	18.5
Previous 12 months^*∗*^	14	13.0
Wheezing		
Lifetime	4	3.7
Previous 12 months	4	3.7
Asthma		
Lifetime	3	2.8
Previous 12 months	2	1.9
Pneumonia		
Lifetime	7	6.5
Previous 12 months	5	4.6
Hospital admission		
Lifetime	18	16.7

^*∗*^ *n* = 107.

**Table 3:** Frequency of referred morbidities in the previous 30 days by children under 5 years of age, Iñapari, 2011.

Variables (*n* = 108)	*n*	%
All morbidities	61	56.5
Fever	31	28.7
Weakness	15	13.9
Headache	7	6.5
*Lack of appetite*	22	20.4
Stomachache^*∗*^	18	16.8
Nausea^*∗*^	4	3.7
Vomiting	17	15.7
Diarrhea	24	22.2
Shortness of breath	4	3.7

^*∗*^ *n* = 107.

**Table 4:** Frequency of referred morbidities in the previous 15 days by children under 5 years of age, Iñapari, 2011.

Variables (*n* = 108)	*n*	(%)
*Morbidities*	**58**	**53.7**
*Respiratory symptoms*	**42**	**38.9**
Wheezing	5	4.6
Runny nose	24	22.2
Dry cough	16	14.8
Productive cough	18	16.7
Sore throat	20	18.5
*Diarrhea* ^*∗*^	**25**	**23.4**
*Stool with blood*	**1**	**0.9**
*Fever*	**25**	**23.1**
*Vomiting*	**17**	**15.7**
*Dehydration*	**3**	**2.8**
*Lack of appetite*	**22**	**20.4**
*Intestinal parasites (“worm”)*	**2**	**1.9**
*Other diseases*	**9**	**8.3**

^*∗*^ *n* = 107.

**Table 5:** Search and access to health services for children under 5 years who with presented morbidities in the last 15 days, Iñapari, 2011.

Variables	*n*	Yes (%)
*Health care seeking behavior during illness*	**36/57**	**63.1**
*Received health care when ill*	**35/36**	**97.2**
*Received drug prescription *	**34/35**	**91.1**
*Drug acquisition*	**34/34**	**100**
Purchased from the public health care system	12/32	37.5
Purchased by the patient at the pharmacy	17/32	53.1
Purchased at the pharmacy by the health service	3/32	9.3

**Table 6:** Factors associated with respiratory symptoms in the previous 15 days in children aged under 5 years, 2011.

Variables (*n* = 108)	Respiratory symptoms			
	*n*	Yes (%)	No (%)	*P* value
Diarrhea in the last 15 days				
No	82	30.5	69.5	*P* = 0.001^*∗∗*^
Yes	25	68.0	32.0	
Type of street				
Soil or no street	83	33.7	66.3	*P* = 0.04^*∗∗*^
Brick or asphalt	25	56.0	44.0	
Type of sidewalk				
Soil or no sidewalk	68	30.9	69.1	*P* = 0.022^*∗∗*^
Brick or cement	40	52.5	47.5	
Maternal smoking				
Never smoked	87	34.5	65.5	*P* = 0.037^*∗*^
Smoker	9	77.8	22.2	
Former smoker	11	45.5	54.5	

^*∗*^Chi-square test, ^*∗∗*^Fisher's exact test.

**Table 7:** Factors associated with diarrhea in the previous 30 days in children aged under 5 years, Iñapari, 2011.

Variables (*n* = 108)	Diarrhea			
	*n*	Yes (%)	No (%)	*P* value
Maternal paid work over the past 90 days				
No	77	27.3	72.7	*P* = 0.05^*∗∗*^
Yes	29	10.3	89.7	
Fever in previous 30 days				
No	77	14.3	85.7	*P* = 0.003^*∗∗*^
Yes	31	41.9	58.1	
Weakness in previous 30 days				
No	93	18.3	81.7	*P* = 0.014^*∗*^
Yes	15	46.7	53.3	
Stomachache in previous 30 days				
No	89	15.7	84.3	*P* = 0.001^*∗*^
Yes	18	50	50	
Vomiting in previous 30 days				
No	91	18.7	81.3	*P* = 0.047^*∗*^
Yes	17	41.2	58.2	
Piped water supply in household				
No	38	34.2	65.8	*P* = 0.026^*∗∗*^
Yes	70	15.7	84.3	
Source of drinking water				
Various sources	35	11.4	88.6	*P* = 0.009^*∗*^
Only mineral	22	45.5	54.5	
Only public system	51	19.6	80.4	
Boiling water before consumption				
No	49	32.7	67.3	*P* = 0.016^*∗∗*^
Yes	59	13.6	86.4	
Type of toilet				
Flushed toilet	35	11.4	88.6	*P* = 0.049^*∗∗*^
Latrine or no toilet	73	27.4	72.6	

^*∗*^Chi-square test, ^*∗∗*^Fisher's exact test.

**Table 8:** Factors associated with hospitalization.

Variables (*n* = 108)	Hospitalization			
	*n*	Yes (%)	No (%)	*P* value
Sex				
Male	58	24.1	75.9	*P* = 0.022^*∗∗*^
Female	50	8.0	92.0	
Age				
<2 y.o	51	5.9	94.1	*P* = 0.004^*∗∗*^
≥2 y.o	57	26.3	73.7	
Years of maternal schooling^1^				
None	2	100.0	0.0	*P* = 0.016^*∗*^
1 to 4 years	6	16.7	83.3	
5 to 8 years	25	20.0	80.0	
8 years	72	13.9	86.1	
Morbidities during pregnancy^1^				
No	44	27.3	72.7	*P* = 0.019^*∗∗*^
Yes	61	9.8	90.2	
Routine health care appointment with medical doctor or nurse in 2010				
No	45	26.7	73.3	*P* = 0.019^*∗∗*^
Yes	63	9.5	90.5	
Child health care appointments				
No	13	38.5	61.5	*P* = 0.011^*∗*^
Yes, once in a while	26	7.7	92.3	
Yes, frequently	28	3.6	96.4	
Only when sick	41	24.4	75.6	

^1^ *n* = 105.

^*∗*^Chi-square test.

^*∗∗*^Fisher exact test.
